# Circulating Endothelial Microparticles in Diabetes Mellitus

**DOI:** 10.1155/2010/250476

**Published:** 2010-06-16

**Authors:** A. F. Tramontano, R. Lyubarova, J. Tsiakos, T. Palaia, J. R. DeLeon, L. Ragolia

**Affiliations:** ^1^Division of Cardiology, Fletcher Allen Health Care and the University of Vermont School of Medicine, Burlington, VT 05401, USA; ^2^Division of Cardiology, Department of Medicine, Albany Medical Center, Albany, NY 12208, USA; ^3^Division of Cardiology, Stony Brook University School of Medicine at Winthrop-University Hospital, Mineola, NY 11501, USA; ^4^Vascular Biology Institute, Stony Brook University School of Medicine at Winthrop-University Hospital, Mineola, NY 11501, USA

## Abstract

*Background*. Endothelial Microparticles (EMPs) are small vesicles shed from activated or apoptotic endothelial cells and involved in cellular cross-talk. Whether EMP immunophenotypes vary according to stimulus in Diabetes Mellitus (DM) is not known. We studied the cellular adhesion molecule (CAM) profile of circulating EMPs in patients with and without Diabetes Mellitus type 2, who were undergoing elective cardiac catheterization. *Methods and Results*. EMPs were analyzed by flow cytometry. The absolute median number of EMPs (EMPs/*μ*L) specific for CD31, CD105, and CD106 was significantly increased in the DM population. The ratio of CD62E/CD31 EMP populations reflected an apoptotic process. *Conclusion*. Circulating CD31+, CD105+, and CD106+ EMPs were significantly elevated in patients with DM. EMPs were the only independent predictors of DM in our study cohort. In addition, the EMP immunophenotype reflected an apoptotic process. Circulating EMPs may provide new options for risk assessment.

## 1. Introduction

Endothelial microparticles (EMPs) are an emerging marker of endothelial cell (EC) dysfunction, and their circulating numbers are elevated in a number of pathologic states including cardiovascular disease [1]. EMPs contain membrane, cytoplasmic, and nuclear constituents, characteristic of their precursor cells that confer to EMPs the properties of circulating multifunctional effectors, promoting inflammation of the arterial wall and thrombogenicity through cellular cross-talk [2]. Leukocyte adhesion and migration are dependent on a range of cellular adhesion molecules (CAMs) that are up regulated in the endothelium during atherosclerosis [3]. Because microparticles are fragments of EC membranes, they also express CAMs. MiRNAs are also contained in circulating microparticles (MPs) and may influence vascular homeostasis [4]. Additionally, studies demonstrate that, EMPs correlate *in vivo* with indices of EC dysfunction, the presence of coronary artery disease and with the complications of Diabetes Mellitus type 2 (DM) [5, 6]. 

DM is a major risk factor for cardiovascular morbidity and mortality, and the proportion of cardiovascular disease attributable to DM has increased over the past 50 years [7]. It is well established that endothelial dysfunction and inflammation are key features of DM and are independent of other cardiovascular risk factors [8]. In type I DM the procoagulant potential of MPs is correlated with the degree of glycemic control, and elevated EMP levels are predictive for the presence and severity of coronary artery lesions [6, 9]. Plaque rupture and acute coronary thrombosis may also be associated with the activation of tissue factor from its encrypted form on MPs [10, 11]. In addition, plaque stability and high risk coronary lesions correlate with the level of circulating EMPs and the expression of specific CD antigens [12]. 

Studies suggest that MPs produced via activating stimuli have different protein expression patterns from those produced via apoptosis. The present study addresses the hypothesis that DM produces EMPs that vary in immunophenotype. Using a flow cytometric approach, we compared the CAM composition of different populations of EMPs isolated from DM patients who were undergoing elective cardiac catheterization.

## 2. Methods

### 2.1. Clinical Study Population

We performed a cross-sectional study that prospectively and sequentially included 40 patients (20 diabetics and 20 nondiabetics) undergoing cardiac catheterization. All catheterization procedures were elective and for suspected coronary artery disease. The following clinical information was collected from all study subjects: age, sex, body mass index, blood pressure, total cholesterol, blood glucose, and history of smoking. Diabetes was defined by a fasting plasma glucose ≥126 mg/dL (7.0 mmol/L), nonfasting plasma glucose ≥200 mg/dL (11.1 mmol/L), or the individual currently being treated with insulin or an oral hypoglycemic agent. BMI was calculated as the ratio of weight-to-height squared; resting brachial blood pressure was measured by an automated sphygmomanometer; and hypertension (HTN) was defined as a resting blood pressure >140/90 mmHg and/or the presence of antihypertensive treatment. Blood lipids and glucose were measured by enzymatic methods, and hypercholesterolemia was defined by total cholesterol >200 mg/dL and/or the presence of lipid lowering drug treatment (NCEP Circ 2002 106). Current smoking was defined as daily consumption of ≥1 cigarette daily for ≥3 months. All patients were fasting for >12 hours. Recent laboratory data and drug therapy were obtained from chart review. The study was approved by the Winthrop-University Hospital IRB committee. 

### 2.2. Blood Sampling

Arterial blood was collected via femoral artery catheter into 3.2% trisodium citrate (Becton Dickinson, San Jose CA). Platelet-free plasma was obtained and subjected to centrifugation at 13,000 g for 45 minutes. The microparticle pellets were resuspended in 100 *μ*L of PBS and stored at −80°C until use in flow cytometry experiments. Samples were processed within one hour after collection and freeze-thawed only once immediately before flow cytometric analysis. 

### 2.3. Electron Microscopy

To assess whether intact MPs were present after plasma preparation, the total number of pellet-derived MPs was assessed with electron microscopy. Pellet-derived MPs were fixed in 4% glutaraldehyde, 0.1 M sodium cacodylate buffer, pH 7.4, postfixed in 1% buffered osmium tetroxide, dehydrated in a graded series of ethanol, and infiltrated in LX112 Epon Resin purchased from Ladd (Burlington, VT). Thin sections were picked up on copper formvar-coated grids, stained with lead citrate and uranyl acetate, and analyzed on a Zeiss EM10 transmission electron microscope.

### 2.4. Flow Cytometric Analysis

Briefly, 3 *μ*L of a flurochrome-conjugated monoclonal antibody against one of the listed CAMs (Table 1) plus 50 *μ*L of prepared plasma were added to tubes (2 antibodies/tube) that were preloaded with fluorescent TruCountTM bead lyophilized pellets (Becton Dickinson Biosciences, San Jose, CA), as outlined previously [13]. Calibration beads were obtained from Molecular Probes (Eugene, OR). Monoclonal antihuman CD62E-FITC, CD105-FITC, CD106-FITC, CD144-FITC, CD31-FITC, CD41a-PE, and CD45-PE antibodies were obtained from Ancell Co. (Bayport, MN). Analysis of EMPs was performed using an FACS Canto Flow Cytometer (Becton Dickinson Biosciences, San Jose, CA), operated at medium flow-rate setting, with log gain on light scatter and fluorescence. Events with 0.2 to 1.0 *μ*m size on FS-SS graph were gated as EMPs. Data from 5,000 events were acquired and analyzed with the use of BD FACSDiva (version 4.1.2, Becton Dickinson). All flow cytometry experiments included EMP samples labeled with single antibody conjugates incorporating the relevant fluorochromes for compensation. The absolute number of EMPs was enumerated from the appropriate dot plot values entered into the following formula:

(1) $$\begin{array}{cc} & \text{Absolute number of EMPs/mL}\\ & \;=\#\text{ of events in EMP region }\left(R2\right)\times \text{total }\#\text{ of beads per tube}/\\ & \;\#\text{ of beads collected }\left(R1\right)\times \text{test volume }\left(50\;\mu \text{L}\right). \end{array}$$

The total number of beads per tube is supplied by the manufacturer and varies among lot numbers. To access the method's ability to distinguish EMPs from platelet MPs, known quantities of pure EMPs from TNF-*α*-activated human umbilical vein endothelial cell culture and platelet MPs from isolated platelets from normal subjects were mixed together. Counts of EMPs and platelet MPs of the mixture were the same as when measured independently. These results support the specificity of the assay for detecting EMPs. To test whether leukocyte MPs might be confused with EMPs, the pan leukocyte marker anti-CD45 was used. Negligible CD31+/CD41a+ EMPs were detected. No significant nonspecific binding of leukocyte MPs was noted.

### 2.5. IL-1 Assay

The concentration of IL-1*β* was measured by bead array (BioRad Cat# 171B12832) using the BioPlex200 (BioRad, Hercules, CA). Briefly, plasma was diluted 1  :  1 in commercial diluent and processed according to the manufacturer's instructions. Unknown concentrations were obtained by extrapolation from a curve of known standards.

### 2.6. Statistical Analysis

Continuous variables were tested for normal distribution with the Kolmogorov-Smirnov test. The Wilcoxon Rank Sum test was used to compare the study variables between DM patients and nondiabetics. Correlation analyses were performed using the Spearman rank coefficient. Multiple linear regression analysis was performed where indicated in order to identify independent variables influencing the prediction of EMP changes in peripheral blood. EMP numbers and data that were not normally distributed are expressed as medians (interquartile range or range). Statistical significance was assumed when a null hypothesis could be rejected at a *P* < .05.

## 3. Results

### 3.1. Subject Characteristics

Subject demographics and clinical characteristics of DM patients and controls are shown in Table 2. DM patients had significantly higher BMIs and fasting plasma glucose. A significant difference also existed in age between the two groups.

### 3.2. Electron Microscopy

**Figure 1:**
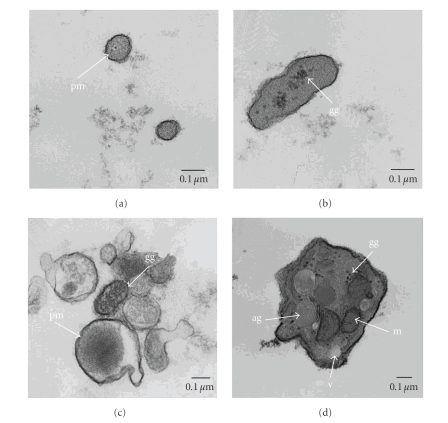
*Various sized microparticles were isolated from platelet-free plasma from a normal subject and analyzed by transmission electron microscopy.* (a–d) Smallest particles noted around 0.2 *μ*m in size. Note intact plasma membrane (pm) and various intracellular organelle remnants including Platelet microparticle (d). Note abundance of glycogen granules (gg), mitochondria (m), vacuole (v), and alpha granules (ag). Scale bar = 0.1 *μ*m.

Plasma MPs are predominately derived from platelets, erythrocytes, and monocytes, with EMPs comprising the smallest circulating MP population [14, 15]. Electron microscopy can be used for better characterization of morphological features and visualization of MPs. MP size varies between 0.2 and 1.0 *μ*m [16, 17]. We performed transmission electron microscopy on platelet-free plasma-derived pellets from both diabetic and nondiabetic patients. Transmission electron micrographs of platelet-free plasma pellet fractions at low and high magnification demonstrated intact MPs of various sizes with cellular organelle remnants (Figure 1). There were no morphological or structural differences of MPs visualized between diabetic and nondiabetic subjects. 

### 3.3. Numbers and Immunophenotype of Circulating EMPs

**Figure 2:**
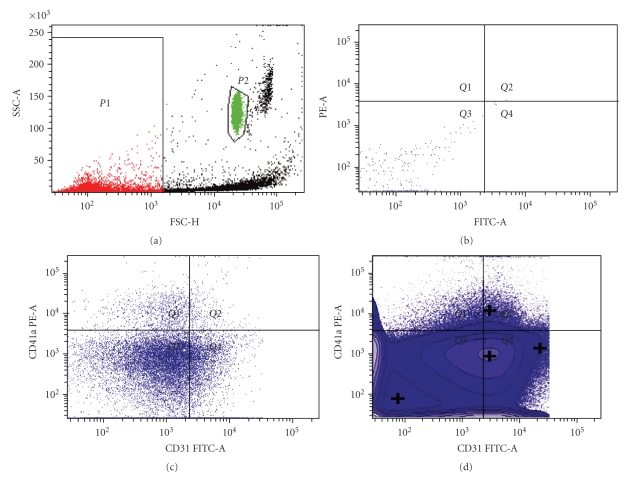
*Gating protocol.* (a) Bivariant analysis efficiently distinguished EMPs from quantitation beads. (b) EMPs from activated EC culture had negligible autofluorescence on FITC/PE channels. (c) Two-color staining with CD41a enables CD31+ EMPs to be segregated from platelet microparticles. (d) Logicle displays provide improved representation of the EMP population with minimal fluorescence.

**Figure 3:**
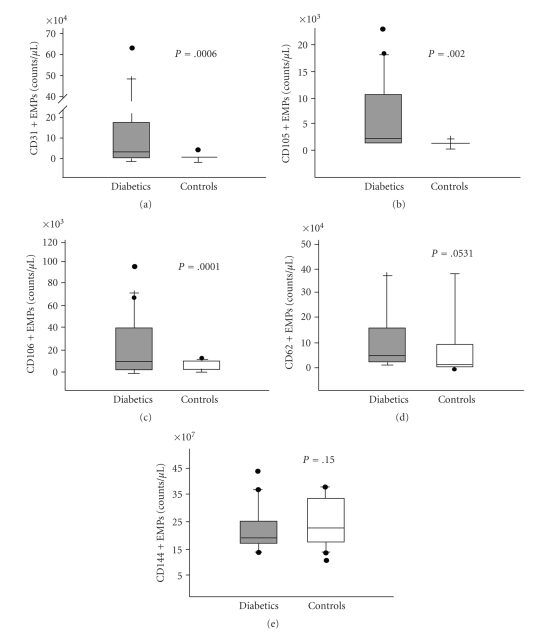
*Diabetic patients had significantly increased absolute number EMPs compared to controls.* Box-and-whisker plot showing plasma values in patients with and without DM: CD31+/CD41− EMP (a), CD 105+ EMP (b), and CD 106+ EMP (c). In these plots, lines within boxes represent median values; the upper and lower lines of the boxes represent the 25th and 75th percentiles, respectively; the upper and lower bars outside the boxes represent the 90th and 10th percentiles, respectively.

EMPs may express adhesion molecules specific for mature endothelial cells (Table 1): examples are CD62E (E-selectin), CD62P (P-selectin), and CD31. Because CD31 is also expressed by platelet-derived MPs, EMP specificity was ensured by the CD31+/CD41− phenotype (CD41 being the platelet gpIIbIIIa complex found on platelets, megakaryocytes, and monocytes). EMPs also exhibit endothelial cell-specific antigens such as CD105 (endoglin, a proliferation-associated protein) and CD144 (VE-cadherin). In both patients and controls, platelet microparticles (PMP) constituted the largest proportion of total MPs. Comparable but low numbers of leukocyte-derived MPs were found in both populations. A known count of larger beads (TruCount beads) acted as an internal standard and enabled us to calculate the absolute number of EMPs per analyzed volume of specimen (Figure 2). We found that the absolute median number of EMPs specific for CD31, CD105, and CD106 was significantly increased in the DM population (CD31+/41a− EMPs: 22238 to 157 × 10^4^/*μ*L [DM versus control], *P* =.0006; CD105+ EMPs: 2200 to 390 × 10^3^/*μ*L, *P* =.002; CD106+ EMPs: 4939 to 740 × 10^3^/*μ*L, *P* = .001) (Figure 3). DM subjects also had elevated levels of CD62E+ EMPs; however this was not significant. There was no significant difference of CD144+ EMPs noted between populations. 

### 3.4. Apoptosis versus Activation

Studies suggest that MPs produced via activating stimuli have different protein expression patterns from those produced via apoptosis; consequently analysis of EMP phenotypic profile may provide clinically useful information on the status of the endothelium. The ratio of CD62E+/CD31+ EMP populations, rather than their absolute numbers, has been described as a criterion for distinguishing activation versus apoptosis. A ratio ≥10 identifies activation while ratio ≤1.0 identifies apoptosis. Our data suggest that apoptosis is an important mechanism for EMP release in the DM population.

### 3.5. Association of EMPs with Components of DM

**Figure 4:**
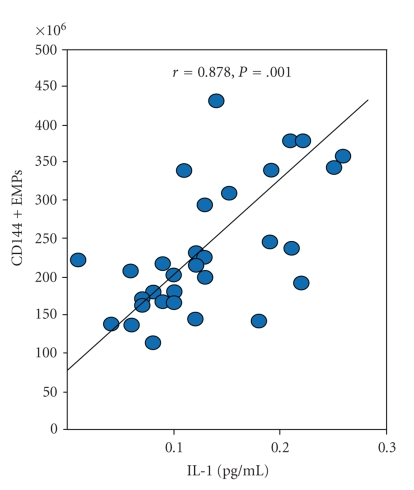
Correlation plot and linear regression line between CD144+ EMPs and the cytokine IL-1.

The release of EMPs into circulation makes their levels closely associated with the degree of vascular damage. To identify independent predictors for EMP elevation, we performed a multivariate linear regression analysis including age, gender, BMI, HTN, plasma cholesterol level, glucose, and EMP phenotypes as independent variables. BMI levels tended to be independently associated with CD31+ EMPs; however, this relationship was not significant. There was a positive correlation between IL-1 levels and the number of CD 144+ EMPs (Figure 4). 

There was no significant difference in IL-1 levels between the diabetic and nondiabetic group.

## 4. Discussion

We demonstrated that DM is associated with increased levels of circulating EMPs. This corroborates previous studies where levels of EMPs were associated with microalbuminuria and microvascular complications in patients with type I diabetes, suggesting that EMPs could be a marker of diabetes-associated endothelial dysfunction [18, 19]. EMPs make up just a small portion of the total circulating population of microparticles. Utilizing electron microscopy we confirmed the presence of intact MPs with various intracellular organelle remnants found in prepared plasma. Exosomes and larger MPs (>1.0 *μ*m in size and often platelets, MP aggregates, or apoptotic bodies) were excluded by our gating protocol. Circulating EMPs are associated with the presence of high-risk angiographic lesions, including eccentric type II, multiple irregular, and lesions with thrombi; however we found no significant relation of EMP levels with the presence or severity of CAD [9, 20]. Multivariate analysis correcting for age, gender, HTN, hyperlipidemia, smoking, and statin use identified EMPs as an independent predictor for the presence of DM. A significant difference was also seen in age, BMI, and blood glucose between the two groups but we did not observe an effect of these variables on EMP levels. In this cross-sectional study of patients undergoing elective cardiac catheterization, we found that circulating levels of CD31+, CD105+, and CD106+ EMPs were significantly increased in patients with DM compared with nondiabetic control patients. Elevated species of EMPs present in diabetic subjects were characterized by a constitutive antigenic immunophenotype suggesting an apoptotic mechanism of EMP generation in diabetic individuals [21]. 

One of the earliest events in atherosclerosis is CAM-mediated adhesion of circulating monocytes to intact activated endothelial cells [22, 23]. Despite the large body of literature on the expression and function of CAMs, the biological properties and function of the circulating form of these molecules remain unclear [24, 25]. Accordingly, analysis of EMP phenotypes provides insight into the nature of endothelial injury. We identified a significant elevation of three EMP immunotypes in diabetic plasma. CD31+, CD105+, and CD106+ EMP subsets are characterized by CAMs integral to the atherothombotic process. Patients with the metabolic syndrome have markedly elevated CD31+ (PECAM-1) EMPs [26]. Additionally, CD31 is an efficient signaling molecule related to angiogenesis, platelet function, thrombosis, mechanosensing of endothelial cell response to fluid shear stress, and regulation of multiple stages of leukocyte migration [27, 28]. Of note, the use of CD31 as a sole marker for EMP detection is controversial and has not been consistently shown to distinguish EMPs from PMPs. CD105 (endoglin) is a transmembrane glycoprotein found in the vast majority of the microvessels in atheroma and with a pronounced expression around the periphery of the lipid core [29]. Vascular cell adhesion molecule-1 (VCAM-1, CD106) is expressed on activated endothelial cells and participates in the inflammatory initiation and progression of atherosclerotic plaques [30, 31]. We also identified elevated levels of 62E+ (E-selectin) EMPs that trended towards significance. E-selectin is also expressed in activated endothelium and was found to be an independent predictor of DM [32]. 

The immunophenotype of MPs depends on whether they are released by cell activation or by apoptotic stimulus [21]. Cellular apoptosis is associated with an increase in cytosolic calcium, with changes in the transmembrane steady state leading to the cleavage of cytoskeleton filaments. These phenomena result in the blebbing and shedding of membrane-derived MPs into the extracellular fluid [3]. In vitro studies have shown that processes of apoptosis are reflected by enhanced expression of constitutive antigens on EMPs (e.g., CD31, CD105) and increased binding of annexin V to EMPs. [33]. In contrast, activation of endothelial cells without apoptosis does not affect the expression of constitutive markers, but significantly increases the levels of inducible antigens on EMPs (e.g., CD62E, CD106) [33, 34]. We found that circulating EMPs from diabetic patients predominately expressed constitutive markers (CD31 and CD105) as opposed to inducible markers. In addition, the ratio of CD62E+/CD31+ populations, rather than their absolute numbers, was used as a criterion for distinguishing activation versus apoptosis. Both of these findings suggest that ECs may release EMPs in an attempt to reverse the apoptotic process by freeing the cell of unwanted signaling molecules like proapoptotic caspase-3 and phosphatatidylserine. Alternatively, membrane shedding could constitute a signaling entity to phagocytes and neighboring cells. These results might be interpreted as an indication of enhanced endothelial cell apoptosis, rather than activation in those having DM. Additionally, some EMP species increase with augmented endothelial dysfunction and inflammation as evidenced in our study by the correlation of CD144+ EMPs with IL-1. 

As biomarkers, EMPs allow access to a typically inaccessible tissue, the endothelium. This study found that EMPs were independently associated with the presence of DM and reflect immunophenotypes important to the atherothombotic process. Therefore, EMPs may be a useful surrogate marker for evaluating endothelial dysfunction and/or injury. There is a paucity of information regarding the change in endothelial function over time as it relates to diabetic control and atherosclerotic burden. To help circumvent these problems studies utilizing serial sampling should be performed in diabetic patients thereby allowing each patient to serve as his own biological control. In addition, the storage and freezing of plasma samples might affect the relative levels of EMPs and the reliability of the results compared to those obtained from freshly isolated preparations. Consequently, protocols need to be optimized for the clinical setting where flow cytometry cannot always be performed on the same day as specimen collection. Caution should also be taken when extrapolating laboratory experiments from the results of clinical studies, because a given EMP type may be present with a variety of compositions and biological behavioral patterns in vitro and in vivo. 

Atherosclerosis is a diffuse, multisystem, and chronic inflammatory disease. Therefore, it is essential to assess total vulnerability burden in subjects with a high likelihood of developing cardiac events [35]. A quantitative method for cumulative risk assessment of DM patients needs to be developed that includes variables based on plaque, blood, and myocardial vulnerability in the outcome. Serum markers like EMPs may be one such marker of “vulnerable blood” in patients at risk of cardiovascular events. Larger prospective studies are needed to establish the incremental value of EMP quantification above established risk markers. Improved knowledge of EMP composition, their biological effects, and the mechanisms leading to their clearance will probably open new therapeutic approaches in the treatment of atherosclerosis in DM.

## Figures and Tables

**Table 1:** EC-associated CAM panel for the analysis of EMPs.

Adhesion molecule	Action	Cell of origin	Expression	Counter-receptor	Target cells
CD62 (E-selectin)	Rolling	Activated EC	Activation	L-Slectin, *β*2 integrins	WBC
CD106 (VCAM-1)	Adhesion	EC	Activation	VLA-4	Mono's, Lymphs
CD105 (Endoglin)	Angiogenesis	EC	Constitutive	CD105	EC
CD31 (PECAM-1)	Adhesion	EC, Pits, WBC	Constitutive	PECAM-1	EC, Pits, WBC
CD144 (VE-cadherin)	Adhesion	EC	Constitutive	CD144	EC

**Table 2:** The clinical characteristics of the 40 enrolled patients.

Parameter	Diabetic	Nondiabetic	*P*-value
	(*n* = 20)	(*n* = 20)	
Age (years)	63.7 ± 11.4	70.8 ± 11.5	.32
Sex			
18 F (45%)	10	8	.75
22 M (55%)	10	12	.37
BMI (kg/m^2^)	33.5 ± 8.6	27.5 ± 5.1	.02
Hypertension	16 (80%)	16 (80%)	.52
Blood Glucose	142.9 ± 55.1	105.8 ± 27	.0067
Total Cholesterol (mg/dL)	163 ± 37.9	176 ± 48.8	.49
Lipid lowering therapy	16 (80%)	15 (75%)	.99
CAD	19 (95%)	15 (75%)	.18
Current smoking	2 (15%)	1 (5%)	.60
